# CrystalPilot: A machine-learning based software platform for single crystal neutron diffraction experiments

**DOI:** 10.1063/4.0000915

**Published:** 2025-10-27

**Authors:** Zhongcan Xiao, Guannan Zhang, Zachary Morgan, Viktor Reshniak, Xiaoping Wang

**Affiliations:** Oak Ridge National Laboratory, Oak Ridge, TN, USA

## Abstract

We present an in-house software platform CrystalPilot, designed to enable smart, adaptive single crystal neutron diffraction experiments at the TOPAZ beamline at Oak Ridge National Laboratory. It leverages machine learning methods to provide intelligent experiment steering and advanced algorithms for background noise treatment.

Neutron scattering experiments are inherently high-cost; therefore, automatic steering is essential for optimizing efficiency and maximizing scientific output. CrystalPilot employs real-time regression-based prediction models to predict the data quality and combine with automatic decision-making to steer the experiments in single crystal neutron diffraction. This approach minimizes the neutron beam time, ensuring experiments remain efficient.

Background noise significantly hampers accurate analysis of material properties. We present two machine learning-based methods for background treatment: KD-Tree Estimation and Ring Feature Extraction. The former use a KD-Tree based data structure to estimate the statistical information of background in different regions, while the latter uses a non-uniform Gaussian fitting model to extract the most prominent aluminum ring artifacts in neutron scattering data. Both methods have been tested and demonstrated to effectively remove background noise, enhancing data quality and making them well-suited for different neutron scattering applications.

By combining these algorithms within a cohesive software platform, CrystalPilot ensures that neutron diffraction experiments remain adaptive to real-time conditions, thereby accelerating experimental workflows and improving data fidelity. This integration of machine learning and statistical modeling establishes a robust framework for neutron experiment steering, significantly enhancing throughput, data quality, and scientific discovery.

